# Developing an Agnostic Risk Prediction Model for Early AKI Detection in Cancer Patients

**DOI:** 10.3390/cancers13164182

**Published:** 2021-08-20

**Authors:** Lauren A. Scanlon, Catherine O’Hara, Alexander Garbett, Matthew Barker-Hewitt, Jorge Barriuso

**Affiliations:** 1 The Christie NHS Foundation Trust, Manchester M20 4BX, UK; catherine.ohara1@nhs.net (C.O.); alex.garbett@nhs.net (A.G.); matthew.barker-hewitt@nhs.net (M.B.-H.); 2 Division of Cancer Sciences, Manchester Cancer Research Centre, The University of Manchester, Manchester M13 9PL, UK

**Keywords:** acute kidney injury, artificial intelligence, clinical decision making, early diagnosis, hematologic tests, machine learning, oncology service, hospital, prospective studies, retrospective studies, risk factors

## Abstract

Acute kidney injury (AKI) is a common complication among oncology patients associated with lower remission rates and higher mortality. To reduce the impact of this condition, we aimed to predict AKI earlier than existing tools, to allow clinical intervention before occurrence. We trained a random forest model on 597,403 routinely collected blood test results from 48,865 patients undergoing cancer treatment at The Christie NHS Foundation Trust between January 2017 and May 2020, to identify AKI events upcoming in the next 30 days. AKI risk levels were assigned to upcoming AKI events and tested through a prospective analysis between June and August 2020. The trained model gave an AUROC of 0.881 (95% CI 0.878–0.883), when assessing predictions per blood test for AKI occurrences within 30 days. Assigning risk levels and testing the model through prospective validation from the 1st June to the 31st August identified 73.8% of patients with an AKI event before at least one AKI occurrence, 61.2% of AKI occurrences. Our results suggest that around 60% of AKI occurrences experienced by patients undergoing cancer treatment could be identified using routinely collected blood results, allowing clinical remedial action to be taken and disruption to treatment by AKI to be minimised.

## 1. Introduction

Acute kidney injury (AKI) is a common complication in hospital inpatients. The annual number of excess inpatient deaths associated with AKI in England is estimated to be above 40,000 and the annual cost of AKI-related inpatient care to be around GBP 1.02 billion, just over 1% of the NHS budget [1].

Patients with cancer are among the highest risk groups of developing AKI, occurring in 7.5–9.5% of the cancer population [2,3]. In addition to the direct impact of AKI on any ongoing treatment, AKIs are associated with lower remission rates and higher mortality [4]. At particular risk are cancer patients enrolled in clinical trials and those presenting with progressive disease who have received previous treatments that cause systemic and kidney stress.

NHS England uses an established method of AKI detection based on the amount of serum creatinine in the blood compared to a patient’s previous creatinine measurement or a baseline for their age and weight. This gives an indicator of how well the kidneys are performing in clearing creatinine from the bloodstream and is currently perceived to be the most accurate method of AKI detection at the time of onset [5,6].

A number of studies have attempted to improve this method, using AI approaches, in particular machine learning, incorporating a large amount of data along with mathematical and statistical approaches to mimic human decision making [7]. These studies sought to predict the occurrence of AKI before onset using methods such as gradient boosted trees [8] and neural networks [9], validating these against established methods of AKI detection including the NHS algorithm and KDIGO guidelines [5,10]. The power of the use of biomarkers from blood samples alongside clinical characteristics in assessing AKI risk has also been demonstrated [11] suggesting the possibility of a machine learning tool for AKI risk from blood result data.

While largely successful in predictive power, these earlier AKI prediction studies were limited by their retrospective nature, lack of prospective or external validation, lack of impact analysis or evidence of clinical and automated implementation [12,13]. Another important consideration is the need for widely collected features to be utilised in machine learning models in order to make external validation feasible [12]. However, where one machine learning prediction tool AKIPredictor [14] was prospectively validated against physician predictions of AKI, the tool achieved similar performance as physicians for the prediction of AKI levels 2 and 3, with physicians tending to overestimate the risk, demonstrating the value of a machine learning tool to supplement physician insight [15].

There is a clear overarching need for interpretable and accessible models that can be implemented into the clinical setting as part of a mitigation strategy [13]. Our aim was to create a risk prediction model that could be widely applicable and utilised along with clinician insight to allow for the identification of cancer patients most at risk of an AKI event, thus allowing mitigation methods to be put into practice before an AKI event arose. We present an agnostic machine learning model created using biomarkers gathered from commonly implemented blood tests in both inpatients and outpatients at The Christie NHS Foundation Trust, comprising 597,403 blood tests for 48,865 patients. We describe the build, test and prospective analysis over a three-month period on 9913 patients, in the manner we expect it to be utilised in practice.

## 2. Materials and Methods

### 2.1. Ethics

The project received internal approval from the Caldicott panel. The clinical data used in this study were collected from blood analysers, output in a de-identified format. No personal information was included in the dataset to comply with the hospital’s Information Governance and Security policy, which takes into account the Data Protection Act 2018 and Caldicott Principles and complies with the Data Security and Protection Toolkit [16,17,18].

### 2.2. Study Population

Historical blood test results were collected for inpatients and outpatients at The Christie NHS Foundation Trust, UK, from 1st January 2017 to 3rd May 2020, totalling 618,719 blood tests occurrences for 51,869 distinct patients. All blood tests used in the analyses were collected as part of the patients’ standard care. To remain fully agnostic from clinical information outside of that provided by the blood analysers, age and sex were not considered in the development of the model, and therefore, are not reported within the baseline characteristics. The process of the transmission of blood test results to our data warehouse is automatic, requiring no manual data input and so we focus on this high-fidelity data resource for our model, not including additional, potentially lower-fidelity data that could introduce human errors at this stage.

### 2.3. Missing Data Handling

If a particular observation was not available for a patient at a particular time, we carried forward the previous value for that blood test. We then removed any rows with missing values for any observation after this imputation, resulting in a final data set of 597,403 blood tests for 48,865 patients.

### 2.4. Study Design

**Figure 1:**
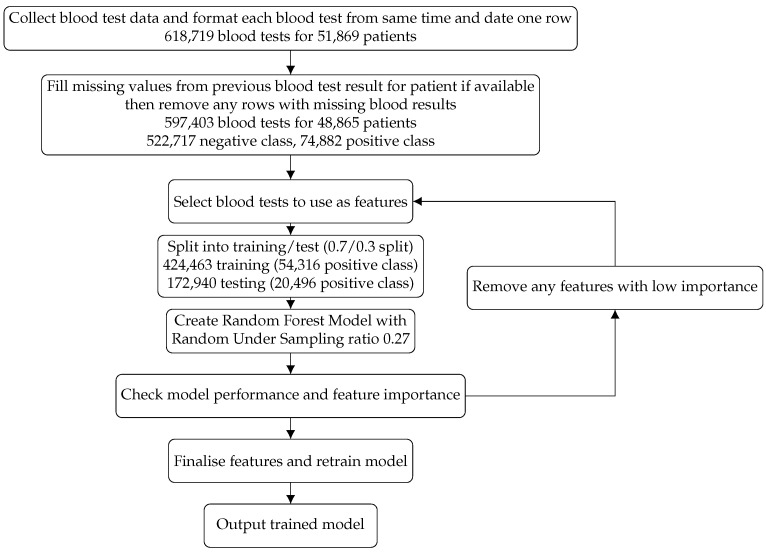
Flow diagram of the data process. The process the data went through from creation to the output model including training/test split and removed data. Feature selection was an iterative process using recursive feature elimination, as shown in the flow diagram.

AKI were detected in our patient dataset using the NHS AKI detection algorithm based on the creatinine level in the blood [5]. The aim was to create a risk prediction model that could be widely utilised and provide enough time for mitigation methods to be put in place. The model was designed to be agnostic of clinical features including age, sex and medical history and aimed to detect the risk of an AKI within the next 30 days solely from blood test results. The nature of the population, cancer patients, used for the development of the model made a 30-day window ideal. In oncology, chemotherapy schedules vary between 7 and 28 days, meaning that the clinician would have multiple opportunities to receive a risk alert based on this prediction from the patient’s routine blood tests for a specific AKI event. This also provides flexibility for the utilisation of these predictions in different health settings. The time between AKI alerts was calculated for each blood test occurrence and each blood test occurrence was then classified as either being within 30 days of an AKI event or not, these becoming the target classes for supervised learning. This gave a split of 522,717 observations that did not lead to an AKI within 30 days (the negative class), and 74,882 that led to an AKI within 30 days (the positive class). A flow chart of the data process is shown in Figure 1.

The features chosen were selected from all available blood tests by first being selected for their availability, maximising the number of results for those blood tests. We then further iteratively selected features using recursive feature elimination [19] in the implementation of the random forest classifier in Scikit-learn [20]. Using this process, a model was created with all available features, the relative importance of each feature evaluated and those contributing least to the model’s decision removed, until removing any additional features was detrimental to the model’s performance by causing a reduction in the area under the receiver operating characteristic curve (AUROC). This method was utilised in order to extract the most important features to model performance in an agnostic way, without incorporating additional knowledge about each feature, and is a method that can be utilised across different models. Details of each feature are given in Table 1.

### 2.5. Selection of Training and Test Samples

The data contained multiple blood results for many patients so, when splitting the data into train and test samples, it was ensured that all results from each patient were either in the training or the test data to minimise leakage based on having seen a result for that patient in the training data. A 0.7/0.3 split was used, modified by this process, resulting in 424,463 results in the training set (54,316 positive class) and 172,940 in the testing set (20,496 positive class). The model used was a random forest classifier implemented in Scikit-learn in Python [20,21] with the random under sampling of the negative majority class implemented by imbalanced-learn [22]. Alternative models were tested but this approach yielded the highest AUROC. The random forest approach utilises a large number of decision trees during the classification process, using the combinations of results from each individual tree to create accurate and stable predictions. The underlying decision tree structure handles complex interactions within the data and aids in model interpretability, with the classifier performing efficiently on large data sets whilst minimising the danger of overfitting [23].

This article was written following the TRIPOD guidelines for prediction model development [24].

## 3. Results

### 3.1. Model Performance

**Figure 2:**
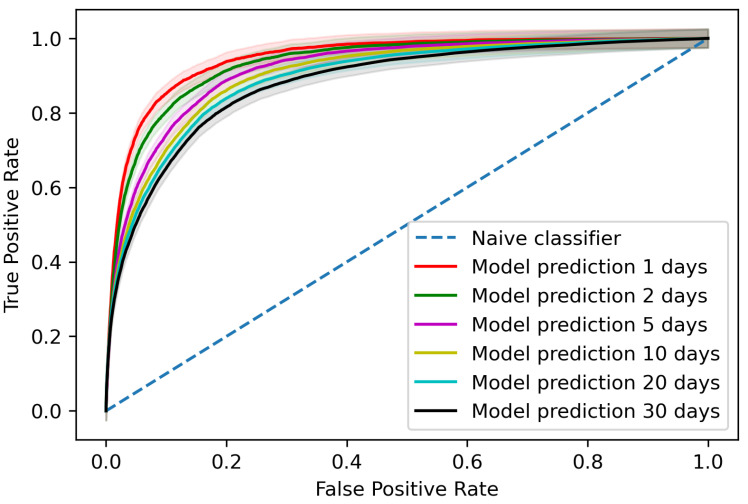
Area under the receiver operating characteristic curve (AUROC) when assessing predictions per blood test. The various lines show the AUROC of 0.881 (95% CI 0.878–0.883) for AKI predictions within 30 days, 0.893 (95% CI 0.891–0.895) within 20 days, 0.903 (95% CI 0.901–0.906) within 10 days, 0.917 (95% CI 0.914–0.920) within 5 days, 0.934 (95% CI 0.930–0.937) within 2 days and 0.947 (95% CI 0.944–0.950) within 1 day, whilst the dashed line shows the comparison to a naive classifier.

The trained model gave an area under the receiver operating characteristic curve (AUROC) of 0.881 (95% confidence interval (CI) 0.878–0.883) when assessing predictions per blood test for AKI occurrences within 30 days of the blood test, as shown in Figure 2 with a comparison for different time steps to AKI occurrence. At the patient level, the trained model detected 65.4% (95% CI 63.2–67.6%) of AKI incidents up to 30 days before AKI occurrence on the testing data with two false positives for every true positive.

### 3.2. Creating Risk Classifications

**Figure 3:**
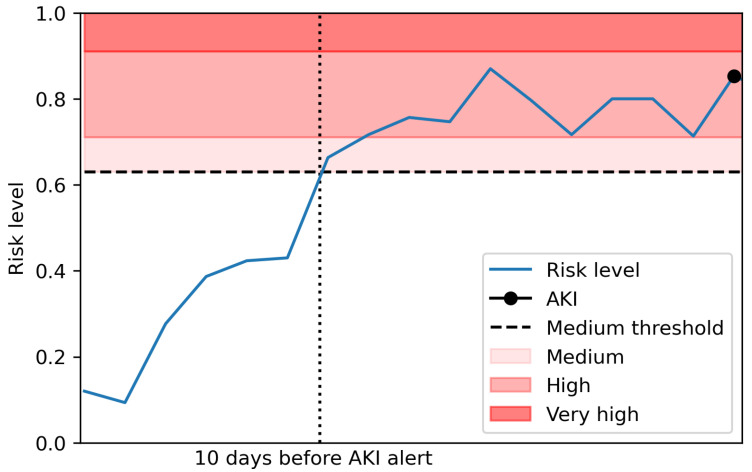
An example of the model’s risk level predictions. The blue line shows the risk level associated with each blood test for a patient, with that above the dashed line denoting a prediction above ‘Medium’. The sections denote ‘Medium’, ‘High’ and ‘Very high’ risk from bottom to top. The plot shows time to the next AKI for the patient with the AKI alert denoted by the black circle.

For the model to be usable in a real setting, the model prediction probabilities were mapped to risk levels given by the proportion of AKI successfully predicted within 30 days. This allows the assignment of a risk level to each blood test in real time, assigning the trained classifier’s prediction probability to one of five risk levels; ’Very low’, ‘Low’, ‘Medium’, ’High’ and ’Very high’. The risk levels were allocated by the proportion of predictions resulting in an AKI within 30 days in each interval as described in Table 2, and a breakdown of the proportion of cases in each risk level given in Table 3. An example of these predictions for a patient is shown in Figure 3.

### 3.3. Prospective Validation

The model was prospectively validated during a three-month period in which risk levels were assigned from the model to blood test results in real time. After 30 days, any AKI alerts for each patient were incorporated. No intervention was put in place during this period and an AKI was deemed to be successfully predicted if the individual received a ‘Medium’, ‘High’ or ‘Very high’ risk level for a blood test up to 30 days before AKI occurrence. This approach was taken to evaluate the performance of the model on out-of- sample data, testing the model’s predictive performance on data that were not available during the model training and test phase in the aim of ensuring the consistency of this performance, with no modification or tuning of the model.

During the period from the 1st June to the 31st August, this approach identified 61.2% of AKI occurrences with just under 1.5 false positives for every true positive, a ratio of 40.7% true positives. During this period, a total of 10,174 patients received blood tests, of which 261 were omitted due to missing data. No patients in this omitted group had an AKI alert within 30 days of any blood test during this period.

For the 9913 patients included in this analysis, 447 had an AKI alert within 30 days of a blood test during this prospective validation period. Risk levels of ‘Medium’ or higher were triggered for 330 of these, resulting in 73.8% of patients with AKI identified before at least one AKI occurrence. There were ’Medium’ or higher risk levels triggered for 484 patients in total during this period and therefore 154 patients who received a risk level of ’Medium’ or higher who did not go on to have an AKI, the false positives.

Breaking this down by month, 242 had an AKI alert within 30 days of a blood test in June, with 146 of these individuals having received a ‘Medium’ or higher risk level, identifying 60.3% of all patients with AKI alerts within 30 days. There were ‘Medium’ or higher risk levels triggered for 221 patients in total during this period.

In July, 233 had an AKI alert within 30 days of a blood test, with 156 of these individuals having received a ‘Medium’ or higher risk level, identifying 66.9% of all patients with AKI alerts within 30 days. There were ‘Medium’ or higher risk levels triggered for 214 patients in total during this period.

In August, 206 had an AKI alert within 30 days of a blood test, with 128 of these individuals having received a ‘Medium’ or higher risk level, identifying 62.1% of all patients with AKI alerts within 30 days. There were ‘Medium’ or higher risk levels triggered for 194 patients in total during this period.

**Figure 4:**
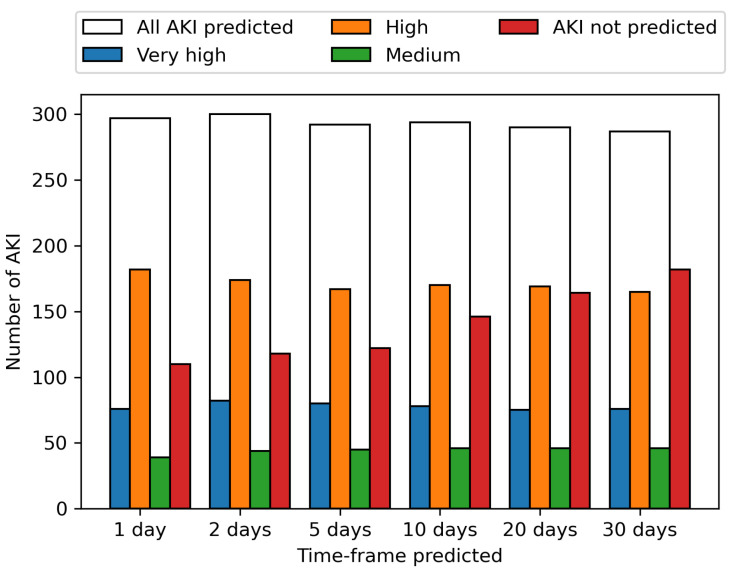
Bar chart showing predicted risk levels at different AKI time-frames. Predicted risk levels at different AKI time-frames during the period from 1st June to 31st August, ranging from 1 day before AKI occurrence to 30 days. The white bar shows all predicted correctly AKIs in the time-frame with the highest risk level predicting this occurrence given by the bars ‘Medium’, ‘High’ or ‘Very high.’ AKI that occurred in the time-frame but were not predicted by a ‘Medium’ or higher risk level are also shown. We see that AKI are more likely to be correctly predicted the closer to incidence with 61.2% of AKI events identified up to 30 days before onset, 63.7% 20 days before onset, 66.8% 10 days before onset, 70.4% 5 days, 71.8% 2 days and 72.8% the day before onset.

These predictions are shown per AKI incidence across the period from the 1st June to the 31st August for various time-frames in Figure 4. The ability to predict an AKI event increases the closer the prediction to AKI incidence is, with 61.2% of AKI events identified up to 30 days before onset, increasing to 63.7% 20 days before onset, 66.8% 10 days before onset, 70.4% 5 days, 71.8% 2 days and 72.8% the day before onset. The prediction of upcoming AKI at the 30-day time-frame allows a wide window for mitigation methods to potentially prevent the AKI, with the increasing likelihood of correctly predicting an AKI the closer the event is, enabling the model to be used in continuous monitoring.

**Figure 5:**
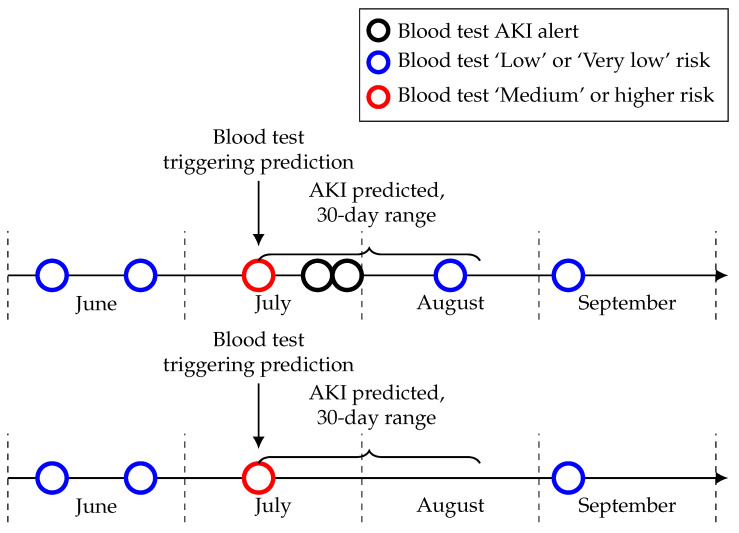
Examples of prediction timelines for patient blood tests. The top image shows an AKI being confirmed after a blood test risk level of ‘Medium’ or higher whilst the bottom one shows an AKI being unable to be confirmed due to a lack of blood tests in the follow up time-frame. Each circle in this figure represents a blood test with the legend denoting a blood test either giving an AKI alert, a ‘Low’ or ‘Very low’ risk level or a ‘Medium’ or higher risk level triggering an AKI prediction.

Among the 75 patients with a ‘Medium’ or higher risk level in June that did not have an AKI alert within 30 days, 39 did not have a blood test within 30 days after the risk level and seven had only one blood test. This could mean they potentially correctly predicted AKI but were never pathologically confirmed (see Figure 5 for a visual example). This proportion is similar across each month with about 50% of those patients with a ‘Medium’ or higher risk level that did not have a confirmed AKI within the time-frame not having another blood test after this risk level. This is something which needs to be considered during the implementation of this model in a clinical setting and suggests that the proportion of seemingly false positives in the risk levels may be lower than it initially appears.

## 4. Discussion

We presented a random forest model to identify acute kidney injury (AKI) before occurrence and earlier than current existing tools. The prospective analysis of our model suggests that around 60% of AKI incidences could be identified up to 30 days before onset, allowing opportunities for mitigation measures to be put in place to prevent or reduce the severity of an event and minimise the disruption to any ongoing cancer treatment. The model uses routinely collected data from blood tests, allowing the prediction procedure to be automated in real time as bloods are collected.

To ensure the model would have the greatest applicability, we used blood test data that were available for as many patients as possible, focusing solely on this high-fidelity resource. Although some patients’ data could still not be included, none in this group developed an AKI within the time period tested. The lower prevalence of AKI in this group suggests that there is a relatively low risk of missing an AKI for a patient that could not be included in the model.

Through this approach, the risk of individual AKI events could be detected before they occurred, allowing clinicians to identify specific ongoing risks to patients as they progress through their treatment. We ensured that the model only used the high-fidelity resource of blood test data which means that the model does not use any of the traditional clinical features that may indicate that the patient is at increased risk of AKI in general. The model we created could be used alongside a separate identification of high risk individuals, and be used for their ongoing monitoring to identify the risk of a specific event occurring, as well as being used in general monitoring for others who may not be known to be at risk. Our model is independent of the underlying circumstances; therefore, it defines a risk based on common features at any time point within the 30 day period.

Although the model provides good early prediction of around 60% of AKI incidences up to 30 days before onset, there are still approximately 1.5 false positives for every true positive, those risk level predictions that are not followed by an AKI within 30 days. Some of these are predictions immediately after an AKI event in the patient, so called ‘trailing predictions’ by Tomašev et al. [9] in their predictive AKI work, which they note can be filtered out in clinical practice. However, we still need to be mindful of these false positives in our clinical implementation, with 484 patients receiving a risk level of ‘Medium’ or higher in our prospective validation, among which 330 had an AKI event after at least one prediction. In clinical implementation, these false positives could cause additional workload for implementers, implementing mitigation strategies that are not necessary for certain patients; however, for those successfully predicted AKI, the benefit from the prevention of AKI, prevention of distress to the patient and reduced workload associated will be larger than the cost of these false positives. In general, the initial pathways leading to AKI in cancer patients are common to the development of AKI in other conditions. Some examples in the oncology field are pre-renal AKI due to dehydration, AKI due to nephrotoxicity by chemotherapeutics, tumour lysis syndrome and post-renal AKI due to ureteric obstructions caused by the disease. All could benefit from early interventions using low-risk interventions, such as encouraging hydration, increasing intravenous fluids’ administration before treatment or performing kidney ultrasound. We also discussed that a proportion of the false positives in our model occurred in patients who did not have another blood test within 30 days of the prediction, potentially meaning there were AKI that were currently not pathologically confirmed. The implementation of this model may aid in detecting and treating AKI that otherwise would never be identified.

In terms of false negatives, some of these resulted from a similar problem to the false positives, in that a patient has not had a blood test in the 30 days prior to the AKI event and so there was no window to predict the AKI before it occurred. Any AKI not predicted by the model would still be detected by the AKI algorithm at time of onset and the patient would receive the appropriate treatment. However, further explorative work needs to be done to further investigate whether there are specific cohorts for which false positives and false negatives are an issue and the potential to account for these by incorporating additional information.

Our model differs from those created by others in the cohort it was defined in, the features utilised and the prediction window. Our model was the first to our knowledge defined in a population of oncology patients and intended to be utilised in both inpatients and outpatients, whereas the models defined by Mohamadlou et al. focused on inpatients and ICU patients, that of Flechet et al. focused on ICU patients and on both inpatients and outpatients in more general populations in the case of Tomašev et al. These models focus on EHR data whereas our model solely focuses on results from routine blood tests. All the models discussed focus on the clinical applicability and the need for predictions with enough time to act but our window of 30 days is the largest time-frame. Given the differences in time-frames, it is difficult to directly compare model performance; however, our model gave an AUROC of 0.881 at 30 days, rising to 0.947 within one day, identifying 61.2% of AKI events in our prospective validation. This compares to an AUROC of 0.921 at 48 h for the model created by Tomašev et al., 0.728 at 72 h rising to 0.872 at onset for the model created by Mohamadlou et al. and 0.82 at 24 h for the model created by Flechet et al., with Tomašev et al. also noting that their model identifies 55.8% of inpatient episodes of AKI and 90.2% of those that require the subsequent administration of dialysis. In terms of model framework, that used by Mohamadlou et al. is most similar to our random forest model, using boosted ensembles of decision trees. Our model performance was slightly improved compared to that of those utilised by others, suggesting the power of the use of features extracted from blood tests when compared to EHR data [8,9,14].

The model is now ready to be implemented through a technology clinical trial to explore the model in practice; the trial will test the value and utility of the model alongside clinical insight to identify patients ahead of AKI events and target possible additional measures. The model is intended to be used alongside traditional AKI detection in addition to clinical expertise and patients will still receive the same standard of care upon AKI detection, but our model will allow for the potential to identify those at risk before onset and allow preventative strategies to be implemented. Our intention is to trial this in a multi-centre setting to demonstrate the utility outside of a cancer centre.

The measure of the success of our model is an expected overall reduction in AKI occurrences. We may also see, at least initially, an effect of an increase in diagnosed AKIs which were previously missed, due to the model predicting AKIs in those that would not have previously had further blood tests scheduled within the 30-day time period and thus not had an AKI confirmed. We may therefore see more patients being diagnosed sooner and receiving treatment whilst in care rather than after discharge or as part of an emergency admission. We also expect to observe an impact of the current COVID-19 pandemic that has led to less hospital attendances and a decrease in the number of blood tests being taken, potentially increasing the amount of undiagnosed AKI.

Future work will also focus on improving the model to identify why some patients are not able to be successfully identified by the current iteration of the model. This could include specific features pertinent to the undiagnosed cohort, through investigative analysis into their shared characteristics. Once interventions start to be put in place, we will be unable to retrain this model in the future to account for any changes in the way blood tests are collected. The intention will then be to create an additional model to better account for those unidentified by this model. In our creation of this model, we solely focused on features from blood test results to predict AKI risk to work in line with the procedure for detecting AKI using creatinine currently utilised at The Christie NHS Foundation Trust and throughout the NHS. The model was created to be used in conjunction with this procedure to facilitate implementation at the Christie and in the wider NHS. However, it has been documented that creatinine rises after AKI [25] occurrence, which may mean the predictions from our model may aid in detecting AKI before severe damage is done. Future work may also explore alternative AKI detection methods and additional biomarkers, for example, including urinalysis to incorporate features known to be markers of AKI and other kidney-related illnesses in order to further improve the predictive performance and potential to prevent AKI.

The unpredicted risk of developing AKI in the cancer population is one of the main problems faced by oncologists in clinical practice. The possibility of patients developing AKI before the start of oncological treatment poses a threat to the outcome of the planned approach to the disease, which could also impact patient–doctor trust. Furthermore, the impact of unexpected AKI in screening to enrol patients in clinical trials is a well-known problem throughout the community of clinical cancer researchers; however, the magnitude of the problem has not previously been studied in depth. Our model, due to its capacity to predict AKI within 30 days, could make it possible to establish actions, through use alongside clinician insight, in an ambulatory setting to prevent AKI events, further hospital admissions and disruption to treatment.

The impact of our AI approach in the oncology field needs to be further described under technology trial conditions. Nevertheless, upon external validation, we envisage an impact on patient well-being, medical and radiation treatment compliance and in clinical trial recruitment.

## Figures and Tables

**Table 1:** Flow diagram of the data process. The process the data went through from creation to the output model including training/test split and removed data. Feature selection was an iterative process using recursive feature elimination as shown in the flow diagram.

Feature	Unit	Mean	Std Dev.
Current CREA/median over the past year	µmol/L	1.01	0.23
Total protein (TP) from blood test	g/L	63.90	8.87
Current CREA/minimum over the past 7 days	µmol/L	1.05	0.17
Current CREA level—mean over the past year	µmol/L	0.03	19.13
Platelet count (PLT) from blood test	×10${}^{9}$/L	251.82	143.17
Hematocrit (HCT) from blood test	L/L	0.35	0.06
Calcium (CA) from blood test	mmol/L	2.27	0.18
Lymphocytes (LYMPH) from blood test	×10${}^{9}$/L	1.53	6.35
Current urea—mean over past year	mmol/L	0.13	1.93
Lactate dehydrogenase (LDH) from blood test	IU/L	406.74	541.05
Red cell count (RBC) from blood test	×10${}^{12}$/L	3.85	0.73
Minimum CREA over the past 7 days	µmol/L	72.29	32.01
White cell count (WBC) from blood test	×10${}^{9}$/L	7.33	9.77
Median CREA over the past year	µmol/L	74.06	30.39
Minimum CREA over the past 2 days	µmol/L	74.18	35.02
Current CREA—CREA 10 days ago	µmol/L	0.02	2.24
Creatinine (CREA) from blood test	µmol/L	75.11	36.18
Current CREA—CREA 20 days ago	µmol/L	0.02	1.03
Abnormal albumin (ALB) 1 if 35 ≤ ALB ≤ 50 2 if ALB ≤ 34 3 if ALB ≥ 51	1, 2 or 3	1: 80.1% 2: 19.6% 3: 0.3%	
Current CREA—minimum over the past 2 days	µmol/L	0.93	5.35

**Table 2:** Mapping model probabilities to risk levels. Predictions from the trained random forest classifier are allocated to 5 risk levels; ‘Very low,’ ‘Low,’ ‘Medium,’ ‘High’ and ‘Very high’ based on the classifier’s prediction probability (p). The risk levels were allocated due to the proportion of predictions resulting in an AKI in the ‘Definition’ column for the interval in the ‘Model threshold’ column.

Risk Level	Definition	Model Threshold
Very high	Over 90% have an AKI within 30 days	0.91<p≤1
High	Over 70% have an AKI within 30 days	0.71<p≤0.91
Medium	Around 50% have an AKI within 30 days	0.63<p≤0.71
Low	Over 70% do not have an AKI within 30 days	0.36<p≤0.63
Very low	Over 90% do not have an AKI within 30 days	0≤p≤0.36

**Table 3:** Proportion of predictions at each risk level for training, test and prospective data. We see that the proportions are fairly consistent across the testing and prospective data but vary from the training data. The proportions for the ‘Very low’ risk level are consistent across the three samples, suggesting that the performance of the model in identifying those at low AKI risk in reality has similar accuracy to the trained case.

Risk Level	Training Data	Testing Data	Prospective Data
Very high	3.16%	0.99%	1.45%
High	6.14%	4.01%	4.74%
Medium	2.10%	2.24%	2.26%
Low	6.72%	11.50%	10.59%
Very low	81.88%	81.36%	80.97%

## Data Availability

The data underlying this article were accessed from The Christie NHS Foundation Trust. We cannot share these data under Section 43(2) of the Freedom of Information Act 2000. Questions or queries will be responded to individually by the corresponding author.

## Abbreviations

The following abbreviations are used in this manuscript:

AKI	Acute Kidney injury
CI	Confidence Interval
AUROC	Area under the Receive Operator Characteristic
EHR	Electronic Health Record
